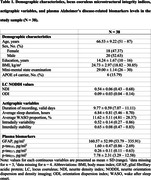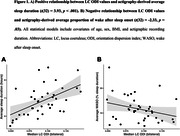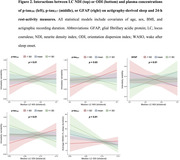# Relationships between locus coeruleus microstructural integrity, sleep, and 24‐h rest‐activity rhythm in the context of plasma markers of Alzheimer’s disease: a 7T MRI study of asymptomatic older individuals

**DOI:** 10.1002/alz.085381

**Published:** 2025-01-03

**Authors:** Maxime Van Egroo

**Affiliations:** ^1^ Faculty of Health, Medicine and Life Sciences, School for Mental Health and Neuroscience, Alzheimer Centre Limburg, Maastricht University, Maastricht, The Netherlands, Maastricht, Netherlands; Gordon Center for Medical Imaging, Massachusetts General Hospital/Harvard Medical School, Boston, MA USA

## Abstract

**Background:**

The brainstem locus coeruleus (LC) is among the first sites of Alzheimer’s disease (AD) pathology, accruing hyperphosphorylated tau as early as in young adulthood. Animal studies indicate that the LC is crucially involved in sleep‐wake regulation, a recently established factor contributing to AD‐related pathophysiological processes. However, the associations between LC integrity and sleep‐wake phenotypes in the context of AD pathology remain poorly characterized in humans. Here, we examined the relationships between *in vivo* 7T MRI‐derived LC microstructural integrity and objective measures of sleep or circadian rest‐activity patterns at different levels of AD pathology.

**Method:**

Thirty‐eight cognitively unimpaired older adults (mean age = 66.53±9.22y., 18 females, **Table 1**) underwent dedicated LC imaging combined with whole‐brain neurite orientation dispersion and density imaging (NODDI) at 7T to extract microstructural indices of neurite density (NDI) and orientation dispersion (ODI) within the bilateral LC. In addition, they performed 10 days of actigraphy from which objective measures of sleep (average sleep duration and proportion of wake after sleep onset (WASO%)) and 24‐h rest‐activity rhythm (intradaily variability (IV), interdaily stability (IS)) were computed. Plasma concentrations of GFAP and phosphorylated tau epitopes were derived from blood samples using Single molecule array technology (p‐tau_181_, p‐tau_231_) and the Meso Scale Discovery platform (p‐tau_217_).

**Result:**

Multiple linear regression models adjusted for age, sex, BMI, and actigraphic recording duration showed that higher LC ODI was associated with longer sleep duration (*t*(32) = 3.93, *p*<.001) and lower WASO% (*t*(32) = ‐2.33, *p* = .03, **Figure 1**). Importantly, interaction models revealed a synergistic deleterious effect of displaying lower LC ODI and elevated levels of p‐tau_217_ on WASO% as well as cross‐over interactions between LC NDI or ODI and p‐tau_181_, p‐tau_217_, or GFAP on IV and IS (**Figure 2**).

**Conclusion:**

These preliminary results suggest that poorer LC microstructural integrity is linked to worse proxy measures of sleep quality and circadian rest‐activity patterns, but only in individuals with elevated levels of AD pathology. Our findings therefore provide novel evidence of the involvement of the LC in sleep‐wake phenotypes in older adults in the context of AD pathophysiological processes, and have implications for the identification of individuals at higher risk for AD‐related sleep‐wake disturbances.